# Effects of 1-day e-learning education on perinatal psychological support skills among midwives and perinatal healthcare workers in Japan: a randomised controlled study

**DOI:** 10.1186/s40359-022-00832-6

**Published:** 2022-05-23

**Authors:** Eriko Shinohara, Yukiko Ohashi, Ayako Hada, Yuriko Usui

**Affiliations:** 1 Department of Nursing, School of Medicine, Yokohama City University, 3-9 Fukuura, Kanazawa-ku, Yokohama, Kanagawa 236-0004 Japan; 2 Josai International University, 1 Gumyou, Togane-shi, Chiba 283-8555 Japan; 3 Kitamura Institute of Mental Health Tokyo, A-Tomigaya Riverland House, 2-26-3 Tomigaya, Shibuya-ku, Tokyo, 151-0063 Japan; 4 Department of Midwifery and Women’s Health, Division of Health Sciences and Nursing, Graduate School of Medicine, The University of Tokyo, 7-3-1 Hongo, Bunkyo-ku, Tokyo, 113-0033 Japan

**Keywords:** Empathy, Communication, Education, Randomised controlled trial, Midwifery

## Abstract

**Background:**

Although midwives are expected to play a key role for psychological support throughout perinatal periods, their educational chances are limited. Versatile teaching strategies such as e-learning may be promising in expanding education. The objective of our study was to clarify the effects of an e-learning educational programme on midwives’ empathic communication skills.

**Methods:**

From April 2019 to September 2019, a randomised controlled trial of a 1-day e-learning educational programme on perinatal psychological issues (both perinatal mental health assessment and empathic communication) was conducted to improve empathic communication skills of midwives and perinatal healthcare workers. Two types of measurements (paper-and-pencil multiple-choice test and video-viewing tests of simulated patient) were used to measure the competency of empathic communication skills.

**Results:**

Participants (*N* = 115) were randomly allocated to two groups (Intervention: *n* = 58, Control: *n* = 57). The intervention group was at a significantly higher level for both post-tests of empathic communication skills compared with the control group. Both intervention and control groups showed improvements in acquiring knowledge about perinatal mental health assessments.

**Conclusions:**

The results of our study show that a 1-day e-learning programme helped improve the midwives' empathic communications skills. Therefore, an effective 1-day e-learning educational programme of perinatal mental health will expand opportunity to learn about empathic communication skills for midwives and perinatal healthcare workers.

*Trial registrations*: UMIN000036052.

## Background

The perinatal period is characterised by the onset of various mental health problems due to role changes in women’s lives or hormonal changes [1–3]. In particular, risk factors include a past history of depression or anxiety disorders and psychosocial factors such as lack of social support, partner conflict, and stressful life events [4]. Professional support is necessary for perinatal mental health. Among perinatal health professionals, midwives and other perinatal healthcare workers are situated in an important position to provide psychological assessment and support for perinatal women. The provision of midwifery assessment of psychological well-being and emotional support for various situation such as depression, anxiety, still birth, neonatal death and infant illness are listed as essential competencies by International Confederation of Midwives [5]. Assessment includes knowledge of diagnosis, symptomatology, and epidemiology of mental disorders commonly seen in the perinatal period such as mood, anxiety, psychotic disorders, bonding disorders, and child abuse. Furthermore, there were several types of treatment for psychological support. With regard to depression, there is no predominant difference between the main treatments with regard to effective psychotherapy [6]. Among several treatments for perinatal depression [6–11], psychological counselling is one of the effective methods [12, 13]. Empathic communication is a basis of psychological intervention [14]. There are reports of midwives' empathic communication being effective in providing psychological support to women [15]. A recent review by Coates and Foureur noted that ‘midwives play an important role in the provision of mental health interventions such as counselling’ [16]. In Japan, the suicide rate among pregnant women is reportedly higher than in other countries (8.7 per 100,000 women), and perinatal mental health support is an urgent health issue [17]. In recent years, the national policy has focused on comprehensive mother and child support. In the Japanese perinatal system, midwives, obstetric nurses, and public health nurses are involved in mental health support in the community continuum of support. Midwives care for approximately 90% of pregnant women who are identified as needing perinatal mental health support [18]. In response to the growing demand for care, Japanese midwifery education requirements also include care for perinatal mental health and the ability to respond to this practice [19]. Midwives are expected to play an important role in mental health support. However, there are limited educational opportunities, as well as no validated educational materials.

Despite the importance of such knowledge and skills, little has been studied about an effective and efficient educational system for practicing midwives [15, 20]. In spite of the need, there are various challenges regarding the issue of perinatal mental health support provided by midwives, such as lack of educational resources or training, and time constraints [21]. To solve these problems, web-based e-learning may be a promising candidate as an educational tool. Access to educational content via the internet is simple and the course can be taken anywhere [22]. In the area of mental health, e-learning is used to train many therapists [23], moreover, e-learning educational interventions have been reported to improve therapist competence and to be cost effective [24].

The use of videos of interview scenes is considered effective as an educational tool to enhance psychological support skills. The use of video materials in psychiatric lecture increases satisfaction and long-term retention for students [25]. It can demonstrate near-real ‘pictures’ as to how to assess mental states and how to conduct and intervention interview. As a means of judging the effects of an educational system, paper-and-pencil tests may be suitable for assessing knowledge but not suitable for assessing clinical skills such as empathic communication. The perceived competence or satisfaction with courses for students and clinicians who receive training may not be appropriate as a measure of their clinical skills. For example, Neumann studied the relationship between students’ satisfaction with their courses and their performance and found a positive correlation between the two among engineering students but no correlation among medical students [26]. ‘Rodin and Rodin report a negative correlation between the amount of learning from lecturers and students' evaluation of their teaching performance [27]. These studies cast doubt whether asking about students’ perceived competency would reasonably reflect their actual performance. In addition, whereas communication skills consist of both verbal and non-verbal parts, paper tests are not likely to evaluate non-verbal communication skills. There is a limit to input and/or output non-verbal communication in a paper test. When communicating emotions to the other person, the proportion of verbal information received is 7% when there is a perceived discrepancy between verbal and non-verbal information; reading the words alone is insufficient, as the visual and vocal non-verbal information takes precedence over the verbal information [28]. It is necessary to read back the other person's responses from role-play rather than receiving them from the written version. Hence, role play tests may be more appropriate to evaluate the skills. Here, a student or a clinician has a mock interview with an examiner who takes the role of a patient or client. However, this is very time-consuming and difficult to standardise (i.e. the examiner’s reaction may differ from one test candidate to another). An alternative method may be showing a short-recorded interview session (video-viewing test of simulated patient interviewed by a midwife) and asking to note what the candidate would say to the patient after watching the recording. This video-viewing test is akin to a mock interview examination but more standardised and easier to quantify the candidate’s level of empathic communication.

The primary aim of this study was to evaluate the effects of e-learning education for empathic communication skills. The secondary aims were to assess the effects of e-learning education for empathic communication skills on perinatal mental health knowledge, and attitudes towards support for women in perinatal period.

## Methods

### Study design and procedure

This was an open-label, parallel-arm randomised controlled trial. The materials were developed for e-learning, but to ensure reliable attendance and test reliability, this study was conducted in face-to-face venues. We invited midwives, nurses, and public health nurses who had roles in perinatal care to participate in a 1-day e-learning course of perinatal mental health education. The participants were randomly divided into two groups: Intervention and control. The contents of the e-learning consisted of two parts: perinatal mental health assessment and empathic communication. They viewed the same educational contents, but the timing of measurement was different. The perinatal mental health assessment part was learned in the morning (about two hours) and the empathic communication part in the afternoon (about two hours). The same paper-and-pencil multiple-choice test (empathic communication skills and assessment knowledge), video-viewing test (empathic communication skills), and an attitude test were conducted before the morning session (the pre-tests) in both intervention and control. Same tests were performed in the afternoon (the post-tests). The post-tests were performed before viewing the empathic communication part for the participants in control group and after viewing for the participants in intervention group.

We compared the participants’ level of empathic communication skills between intervention group and control group. The sessions took place in Oita (April 2019), Yokohama (April 2019), and Sendai (June 2019).

### Educational content (Intervention)

The present educational course was developed by the T. and F. Kitamura Foundation of Studies and Skill Advancement in Mental Health (Tokyo). The material was recently developed for clinical midwives, as well as other perinatal health professionals, to acquire knowledge and gain skills of empathic communication in perinatal mental health issues. Both the perinatal mental health assessment and empathic communication sections consist of eight 15-min video scripts. The perinatal mental health assessment section described (a) diagnostic criteria and the importance of mental health assessment, (b) the epidemiology of mental disorders commonly seen in the perinatal period, (c) mood disorders, (d) anxiety disorders, (e) psychotic disorders and puerperal psychosis, (f) child abuse and intimate partner violence, (g) perinatal bonding disorders, and (h) perinatal grief. The empathic communication section described (a) preparation for a mental health interview, (b) basic interview skills, (c) active listening, (d) probe techniques, (e) presentation of a case of acute insomnia after childbirth, and (f) catharsis.

### Participants

Participants were midwives, nurses, and public health nurses who had roles in perinatal care. In Japan, in addition to midwives, obstetric nurses, and public health nurses also provide psychological support to women during the perinatal period. Therefore, such professionals other than midwives were included in this study. Participants were excluded if they had taken the same e-learning course before the study or were aged over 80, since the rate of internet use drops sharply for people over 80 years old, they are considered as difficult target population for e-learning education in Japan [29]. We recruited participants by sending a study recruitment letter to midwife associations, hospitals, clinics, and birth centres in Oita Prefecture, Yokohama City, and Sendai City during periods of February 2019 through May 2019. Those wishing to participate in the study were contacted by the researcher, who then sent them a form explaining the details of the study and consent form. Participants who were included in the study submitted their signed consent form to the researcher on the day of the session.

### Randomisation and masking

Randomisation was performed with a random chart with a block size of four. The randomisation chart was created by a researcher (E.S). A consecutive number of sealed opaque envelopes with a randomisation letter (A or B) were prepared by a research assistant. Envelopes were opened by research assistants, who were not researchers, when the participants came in for the seminar, who were then allocated to one of the two groups. Due to the characteristics of the intervention, masking was not applicable in this trial.

### Outcomes

The primary outcome was improvement of empathic communication skills rated using a video-viewing test of a simulated patient interviewed by a midwife (ECS-V). Secondary outcomes were improvement in the empathic communication skills rated using paper-and-pencil multiple-choice tests (ECS-P), knowledge of perinatal mental health assessment rated using paper-and-pencil multiple-choice tests (KPMHA), and attitudes towards support for women in perinatal periods.

### Measurement

*Demographic information and other variables*: In the pre-test, participants were asked their age, years of clinical experience, educational background, and job title.

*Empathic communication skills*: We used two types of measurements about the participants’ competency to empathically communicate with pregnant women. One was a paper-and-pencil multiple-choice test with five questions to evaluate the empathic communication skills (ECS-P). Here, a short case vignette was presented with four choices for the best empathic response. One point was given for the correct answer whereas the other answers received zero points. The total score ranged from 0 to 5.

The second was a video-viewing test of a simulated patient interviewed by a midwife to evaluate the empathic communication skills (ECS-V). A short (approximately 5 min long) video script (for example see “Appendix”) was shown and was followed by the question ‘If you were in charge of the care of this woman, what would you say to her? Give your response verbatim’. The empathy of each narrative was objectively assessed by the same evaluators (ES, AH, and YU) using the Empathic Understanding in Interpersonal Processes scale (EUIP) [30]. Throughout the study, the allocation was concealed from the researchers. The EUIP has construct validity and has a single item which is rated using a 5-point scale from not empathic at all (1) to very empathic (5). Low scores on the EUIP indicate that the participant’s response is irrelevant to or even antagonising the patient’s emotion while higher scores on the EUIP indicate that their utterance is in accordance with the patient’s emotion or even leading the patient into deeper insight. This evaluation was performed in the pre- and post-tests using different video scripts. In order to check interrater reliability of the EUIP assessment, the three raters independently rated the verbatim responses of 20% of the participants. Interrater agreement was κ = 0.80. In the remaining 80% of cases’ responses were rated by the three raters separately.

*Knowledge of perinatal mental health assessment (KPMHA)*: To assess the knowledge of perinatal mental health, a pencil-and-paper multiple-choice test with 10 questions, each with a choice of four answers, was administered. Questions were created by psychiatrist, obstetricians, midwives, nurses, and clinical psychologists, who were experts in perinatal mental health for evaluation of educational effects of content and this study. Items that test basic knowledge of psychological assessment, which is important in perinatal clinical practice, have been carefully selected by these experts. One point was given for the correct answer whereas the other answers received zero points. The score ranged from 0 to 10. To ensure an equivalent level of difficulty for pre- and post-tests, pilot-tests of 82 questions were conducted among six midwives, who were not eligible to participate, to calculate the rate of correct answers, and 30 questions (15 each for pre- and post-tests) of the same level of difficulty were chosen. The final versions were asked in the pre- and post-tests.

*Attitude for psychological support*: We used the Counsellor Response Form (CRF [31]) to measure the participant’s active attitude towards providing psychological support to the case described in the video script. The CRF has a single item and is rated using a 9-point scale, ‘Do not want support her’ (1) to ‘Want to support her’ (9). This was asked in the pre- and post-tests.

### Ethical considerations

The study protocol was approved by the Institutional Review Board of Tokyo Healthcare University (Kyou30-35C) and registered with UMIN-CTR in Japan (UMIN000036052; dated 1, March 2019). This clinical trial adhered to the Code of Ethics of the World Medical Association and the clinical research ethical guidelines for human subjects established on 27 April 2015, by the Ministry of Education, Culture, Sports, Science and Technology, and the Ministry of Health, Labour and Welfare, Japan.

### Sample size and power

There have been few previous studies of this kind. Referencing a study using the EUIP [32], based on 95% power to detect a significant difference (α = 0.05, two-sided), 164 participants were required.

### Statistical analysis

We used two-way analysis of variances (ANOVAs) to examine the main effects of the group (intervention vs control) and the timing (pre- vs post-tests) and their interactive terms on the scores of ECS-V, ECS-P, KPMHA and attitude. All statistical analyses were conducted using SPSS version 26.0.

## Results

**Fig. 1:**
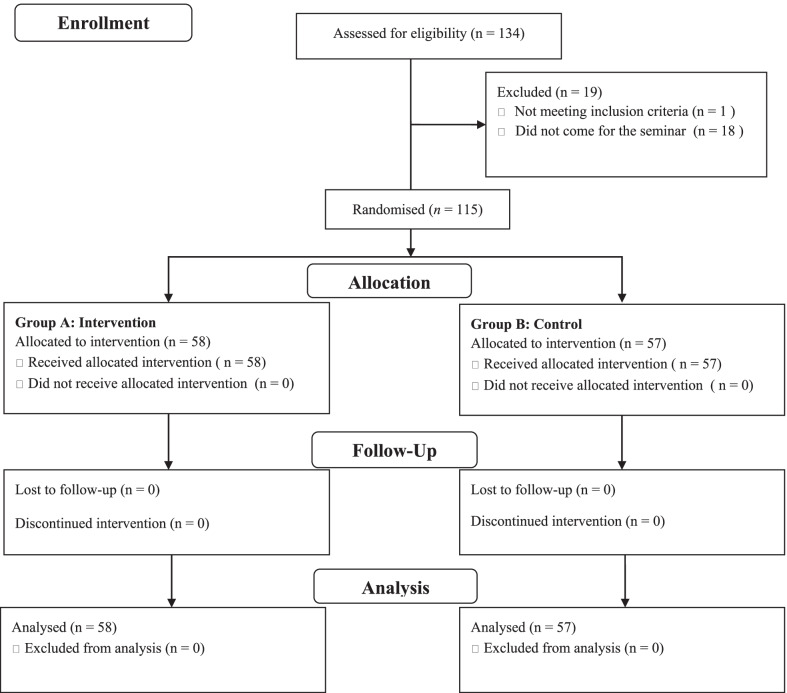
Flow diagram of the trial

Of the 134 who were assessed for eligibility, 115 consented to participate and were then randomised to the intervention (n = 58) and control (n = 57) groups. Figure 1 displays a flow diagram of trial recruitment and follow-up. All participants were followed and included to analysis. Participants were midwives (n = 105), nurses (n = 5), and public health nurses (n = 5). The participants’ job title, age, duration of clinical practice, and education are in Table 1.

**Table 1:** Backgrounds of participants

	Intervention (n = 58) *M* (*SD*) / *n* (*%*)	Control (n = 57) *M* (*SD*) / *n* (*%*)	*p* (95%CI)
*Job title*			.411
Midwife	54 (47.0)	51 (44.3)	
Public health Nurse	1 (2.6)	4 (1.7)	
Obstetric Nurse	3 (0.9)	2 (3.5)	
*Age*	47.3 (12.7)	45.8 (12.9)	.521 (− 3.20 to 6.29)
*Years*	20.1 (11.9)	17.3 (12.5)	.226 (− 1.74 to 7.29)
*Education*			.495
College	28 (24.3)	29 (25.2)	
Junior-college	6 (5.2)	10 (8.7)	
University	16 (13.9)	10 (8.7)	
Graduate school	8 (7.0)	8 (7.0)	

M, mean; SD, standard deviation

The ECS-V and ECS-P were moderately correlated, *r* = 0.409, *p* < 0.001. For the primary outcome, the scores of the ECS-V (EUIP) showed the main effects of the group (*F*(1) = 4.302, *p* < 0.05), timing (*F*(1) = 21.967, *p* < 0.001) as well as the interactive effect (*F*(1,1) = 25.157, *p* < 0.001). For the secondary outcome, the scores of the ECS-P showed the main effects of the group (*F*(1) = 32.364, *p* < 0.0001) and timing (*F*(1) = 32.364, *p* < 0.001). Their interactive effect was also significant (*F*(1,1) = 40.269, *p* < 0.001). Thus, both the ECS-V and ECS-P scores were higher among the intervention group participants at the post-test occasion (Table 2). These findings indicated that the present programme improved the participants’ empathic communications.

**Table 2:** Empathic communication skills, assessment of perinatal mental knowledge and attitude between groups

	Intervention		Control		*p*
	Pre-test *M* (*SD*)	Post-test *M* (*SD*)	Pre-test *M* (*SD*)	Post-test *M* (*SD*)	
ECS-V(EUIP)	1.81 (0.79)	2.82 (0.91)	2.07 (0.86)	2.03 (0.97)	< .001
ECS-P	4.20 (1.12)	5.48 (0.81)	4.49 (0.89)	4.42 (0.76)	< .001
KPMHA	4.07 (1.24)	8.04 (1.12)	3.95 (1.34)	8.05 (0.97)	0.270
Attitude (CRF)	7.96 (1.14)	8.11 (1.03)	8.20 (0.98)	8.09 (1.01)	2.998

M, mean; SD, standard deviation; ECS-V, Empathic Communication Skills (Video-viewing tests of simulated patient interviewed by a midwife); EUIP, Empathic Understanding in Interpersonal Process; ECS-P, Empathic Communication Skills (Paper-and-pencil multiple-choice tests); KPMHA, Knowledge of Perinatal Mental health Assessment (Paper-and-pencil multiple-choice tests); CRF, Counsellor Response Form

The other secondary outcome, KPMHA scores showed the main effect of timing (*F*(1) = 891.081, *p* < 0.001) but no main effect of the group (*F*(1) = 0.092, NS). No interactive terms were significant (*F*(1,1) = 0.270, NS). Therefore, the KPMHA of both the intervention and control groups improved after the course. For attitude, no main effect was observed for either group (*F*(1) = 2.998, NS) or timing (*F*(1,1) = 0.061, NS).

## Discussion

### Main findings

Our results indicated that the 1-day e-learning educational programme of perinatal mental health issues was effective in improving the midwives and perinatal healthcare workers’ empathic communication skills with perinatal women. In addition, this 1-day e-learning programme enhanced the participants’ knowledge about perinatal mental health assessment. As expected, both the intervention and control groups showed improvement in knowledge of perinatal mental health problems whereas only the intervention group showed a marked increase in empathic communication skills. This may support the educational effects of the skills part of the programme. This positive result may come from the design of the educational programme in which the participants learn perinatal mental health assessment before the empathic communication skills. Since knowledge is a factor underlying psychological support attitudes [32], combination of both knowledge and skills may have synergistic effects for empathic communication skills. Another strength of this study is the use of many video scripts describing vignettes of different mental health problems (e.g. mood and anxiety disorders, bonding disorders, and child abuse) as well as role plays of empathic communication interviews. These may give a more realistic picture of clinical knowledge and skills than texts in leaflets and books. Video materials incorporated in psychiatric lectures have been reported to increase students’ satisfaction and long-term retention [25]. Our study also used videos of patients for evaluation of empathic communication skill. Simulated patients are used as a means of evaluation in the objective structured clinical examinations (OSCEs). They are often used in clinical education, and it is thought to be appropriate to assess communication skills [33]. However, OSCEs require staff and time for examinations, limiting staff availability and access. In the present study, the delivery of the e-learning did not require many staff and required limited time for preparation. Therefore, using e-learning for education and evaluation may be more convenient and cost effective than OSCEs.

Research and clinical interest are the only modest correlation between participants’ competency rated by ECS-P and ECS-V. In the former, the participants were requested to select a response which they thought as the most empathic. This is a reflection of mere ‘understanding’ of empathic communication. In the latter, however, they were requested to spontaneously ‘invent’ a verbatim response to an imaginary patient. This is a reflection of their skills of empathic communication. The ECS-V may be more difficult to answer but realistically similar to a clinical setting. Therefore, we think that the ECS-V is a better measure to reflect the participants’ clinical skills of empathic communication.

Midwives feel a lack of skills and confidence for perinatal mental health care [16]. There is demand for learning needs for perinatal mental health and care [34], although one barrier is lack of accessible educational resources [21]. There are few educational research reports specific to psychological support for perinatal mental health. A series of psychological support education seminars for midwives and nurses were effective in a previous study [15]. However, two-days training was not found effective for midwives [20]. Unlike Saito et al.’s study, which was designed as a several-day course, the present study used only 1-day e-leaning material. A recent systematic review reported the most effective duration for intervention was over four hours [35]. We used a four-hour e-learning course and although the duration of our course was shorter than that used in the previous study [15, 20], this course is promising as means of the mental health education for busy perinatal health professionals. The e-learning used in this study substantially improved midwives’ and nurses’ perinatal mental health assessment knowledge as well as empathic communication skills. The e-learning educational tool used in the present study may be promising in delivering education to many experts regardless of time and place in a very short time. Assessment of psychological well-being and emotional support is an essential competency for midwives [5]. In the context of Japanese national policy and midwifery education requirements [19], effective teaching materials for educational effectiveness using e-learning will enable a large scale, low-cost, bottom up of skills acquisition. In addition, the study was originally planned and conducted before the COVID-19 pandemic and did not intend to use e-learning due to COVID-19. However, the COVID-19 pandemic made the use of e-learning increasingly significant.

### Strength and limitation

This was the first randomized controlled trial of e-learning education that specialized in perinatal psychological education. This study has several strengths. Randomisation was implemented to avoid arbitrary groupings and to adjust for potential background bias. In addition, the evaluation of empathic communication was objective, as it was conducted by three experts and inter-raters to ensure credibility, as empathy is considered to be a subjective variable. However, this study did have some limitations. First, the participants responded to our request and were therefore likely to be highly enthusiastic about learning about perinatal mental health issues and psychological support. This may have contributed to the favourable effects of this intervention. Thus, the effects for midwives or nurses with less interest in perinatal mental health issues may have been lower, compared to the estimates of this report. Second, the e-learning material was provided in a 1-day seminar. The results may differ if the course materials were accessed through online mediums by the participants, depending on their convenience. Third, the sample size was smaller than expected. Further research with a larger sample size is required. In addition, the long-term effects of the educational programme are unknown, as it was conducted over a short interval.

In this study, the participants were only clinicians. For further research, since perinatal mental health education is an important topic for midwifery and nursing students, this study should be replicated with undergraduate students.

## Conclusion

The present educational programme improved the participant midwives and perinatal healthcare workers’ empathic communication skills. The 1-day e-learning education of empathic communication skills used in this study was effective and efficient in improving midwives’ supportive skills. Busy clinical midwives are recommended to improve their communication skills in a short duration via e-learning educational materials that are well designed. E-learning education method may pave the way for efficient and effective education about mental health care.

## Data Availability

The datasets used and/or analysed during the current study are available from the corresponding author on reasonable request.

## Appendix: A sample summary of a case vignette for ECS-V

Ms. A. is a 28-year-old nulliparous woman, 18 weeks pregnant, visiting an antenatal clinic. She said, “I was very happy to learn that I was pregnant.” However, her husband was negative saying “I do not really want a kid. We are not financially capable of raising a kid.” In the past month, Ms. A. has been feeling exhausted. She feels pain in the hips and shoulders. She does not feel like doing anything. This continued for days. Things she used to enjoy doing, such as watching TV, were no longer enjoyable. Her mother was concerned and came to check up on her frequently. The mother said, “You’re talking slower than usual, is everything all right?” She felt like she still had nausea and was not hungry at all. During the night, she kept waking up frequently. She said, “I started to feel that I am not fit to be a mother if I kept being this way.” She also added “My mother was abusive … and I do not want to become like my mother.” She spontaneously started talking about her marriage. She married when she was 22 after dating her husband for 3 years. When she realised that she was pregnant the following year, she and her husband decided to go through with an abortion. This is because “my husband had just started a new job and he said, ‘It’s too early for kids.’ And I didn’t want to give up my job at the travel agency.” At that time, “there was no hesitation as I was devoted to my work.”

## Abbreviations

ECS-V: Empatic communication skills using video-viewing test of simulated patient interview by a midwife
ECS-P: Empathic communication skills using paper-and-pencil multiple-choice test
KPMHA: Knowledge of perinatal mental health assessment
EUIP: Empathic Understanding in Interpersonal Processes scale
CRF: Counsellor Response Form
ANOVA: Analysis of variance
OSCE: Objective structured clinical examination
